# Close Correlation between Season of Birth and the Prevalence of Bronchial Asthma in a Taiwanese Population

**DOI:** 10.1371/journal.pone.0080285

**Published:** 2013-11-20

**Authors:** Wei-Chiao Chang, Kuender D. Yang, Man-Tzu Marcie Wu, Ya-Feng Wen, Edward Hsi, Jen-Chieh Chang, You-Meei Lin, Ho-Chang Kuo, Wei-Pin Chang

**Affiliations:** 1 Department of Clinical Pharmacy, School of Pharmacy, Taipei Medical University, Taipei, Taiwan; 2 Cancer Center, Kaohsiung Medical University Hospital, Kaohsiung Medical University, Kaohsiung, Taiwan; 3 Department of Pharmacy, Taipei Medical University-Wan Fang Hospital, Taipei, Taiwan; 4 Master Program for Clinical Pharmacogenomics and Pharmacoproteomics, School of Pharmacy, Taipei Medical University, Taipei, Taiwan; 5 Department of Medical Research and Development, Show Chwan Memorial Hospital in Chang Bing, Changhua, Taiwan; 6 Institute of Clinical Medical Sciences, National Yang Ming University, Taipei, Taiwan; 7 Department of Medical Research, Kaohsiung Medical University Hospital, Kaohsiung, Taiwan; 8 Genomic and Proteomic Core Laboratory, Department of Medical Research, Kaohsiung Chang Gung Memorial Hospital and Chang Gung University College of Medicine, Kaohsiung, Taiwan; 9 Department of Pediatrics, Kaohsiung Chang Gung Memorial Hospital and Chang Gung University College of Medicine, Kaohsiung, Taiwan; 10 Department of Healthcare Management, Yuanpei University, Hsinchu, Taiwan; Cincinnati Children's Hospital Medical Center, United States of America

## Abstract

**Background:**

Bronchial asthma (BA), atopic dermatitis (AD), and allergic rhinitis (AR) are common allergic diseases. Environmental factors were indicated to influence the development of allergic diseases.

**Objective:**

To evaluate the correlation between the month of birth and the prevalence of allergic diseases in Taiwan.

**Methods:**

Data from 104,455 children were collected from the National Insurance Research Database of Taiwan. Subjects were identified by at least two service claims for ambulatory care or one claim for inpatient care. All of the enrolled patients were aged 7∼15 years in 2010. In a bio-clinical data analysis, total immunoglobulin E (IgE) and ImmunoCAP™ allergen data (CAP) from mothers and infants were collected in a medical center in Taiwan. Correlations between children's allergic factors and the season of birth were assessed.

**Results:**

A significant difference in the prevalence of BA according to the month of birth (Χ^2^ = 18.2, *p*<0.001) was found in the Taiwanese population. The fewest schoolchildren with were born in May (7.21%), and the most were born in October (10.59%). However, no tendency for the prevalence of AD (Χ^2^ = 4.6, P = 0.204) or AR (Χ^2^ = 4.3 P = 0.229) was found. In addition, we found that children born in autumn (August to October) had a higher prevalence of BA compared to those born in spring (February to April) (odds ratio: 1.13; 95% confidence interval: 1.05∼1.21). In a bio-clinical data study, markers of maternal and childhood allergies including IgE and CAP were detected in a risk analysis section. Children who were born in autumn had higher levels of CAP and total IgE.

**Conclusions:**

The findings of this study showed that the month of birth was closely correlated with the prevalence of BA and higher levels of CAP and IgE.

## Introduction

Allergic diseases such as allergic rhinitis (AR), bronchial asthma (BA), and atopic dermatitis (AD) are common and increasing in prevalence in developed countries. The pathogenesis of allergic diseases is complex because these disorders are influenced by a combination of genetic and environmental factors [1]. Dysregulated innate immune responses result in acute inflammatory symptoms leading to allergies in susceptible individuals [2]. A number of genetic polymorphisms were identified to associate with susceptibility of allergic diseases [2]–[7]. Additionally, environmental factors such as ambient air pollution and passive smoking were also shown to increase the risk of allergic diseases [8].

Previous studies indicated that being born in winter is associated with a higher incidence of allergic diseases such as atopic dermatitis [9]. This is possibly because birth in winter involves a longer exposure to indoor or domestic allergens, thus increasing the risk of respiratory viral infections. In addition, environmental factors including chemical irritants, seasonal climate changes, and bacterial colonization were reported to associate with allergic diseases such as BA [10]–[14].

In this study, we used data from the National Health Insurance (NHI) Research Database (NHIRD) in Taiwan and tested the correlation between factors of month of birth and allergic diseases (BA, AD, and AR). We also evaluated the association between season of birth and bio-clinical data, such as immunoglobulin E (IgE) and ImmunoCAP™ allergen (CAP), which are related to allergy-like symptoms. We expected to understand the relationship between season of birth and allergic diseases.

## Methods

### 1 Database

We used data from the NHIRD in Taiwan, which is a longitudinal database of one million randomly selected subjects (LH2005). The NHI program was implemented in Taiwan in 1995 to provide comprehensive coverage for medical care. Approximately 98% of the Taiwanese population is enrolled in the NHI program by the end of 2005 [15].

**Figure 1:**
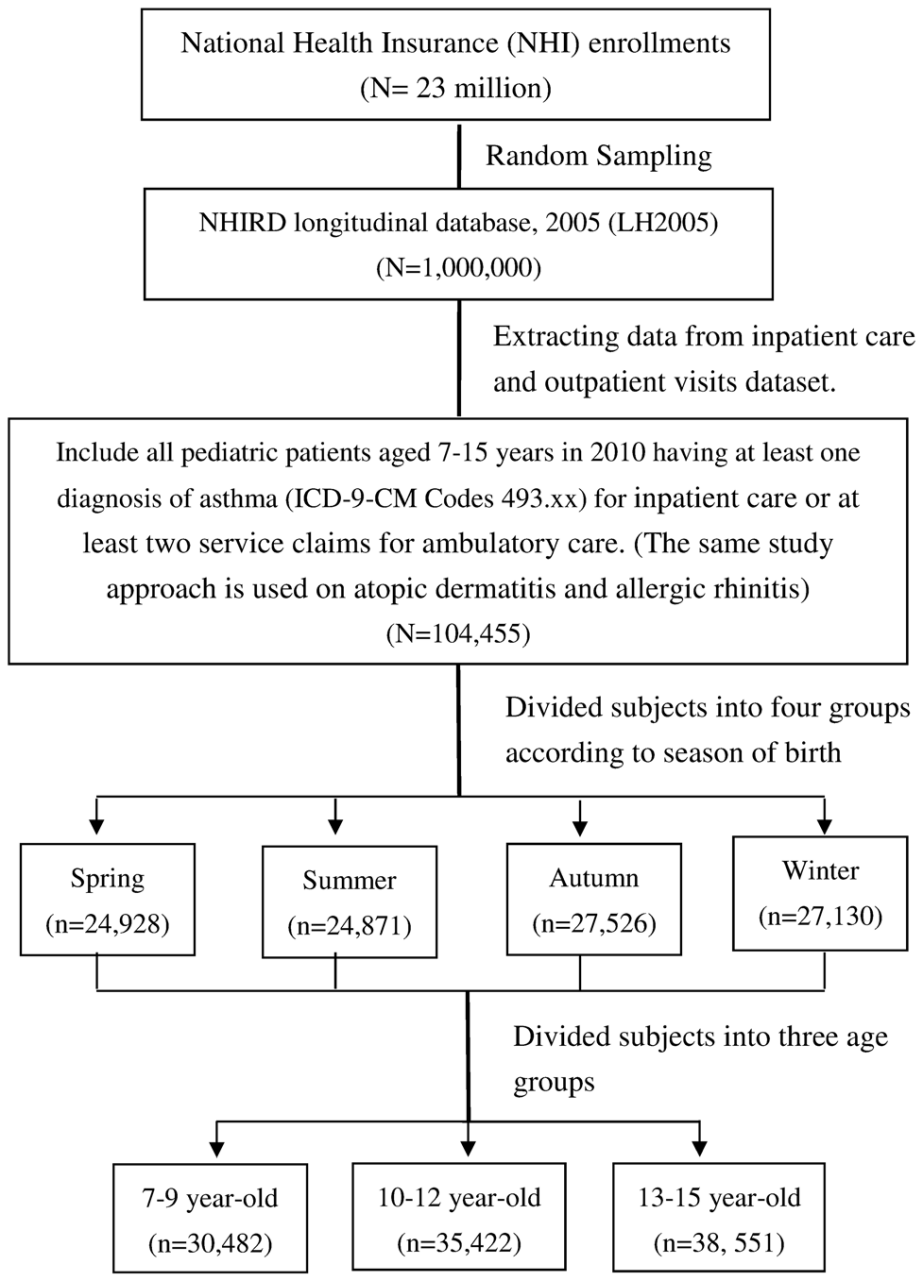
Flow Chart of the Selection of Study Subjects from the National Health Insurance Research Database in Taiwan.

The LH2005 contains the entire medical claims data of one million beneficiaries enrolled in 2005 including scrambled patient identification number, birth date, gender, one principal diagnosis and up to two or four secondary diagnoses for each outpatient and hospitalization claim of the International Classification of Disease, Ninth Revision, Clinical Modification (ICD-9-CM) codes, medications, and date of visit to a medical institution. These data are randomly sampled from the 2005 registry for beneficiaries of the NHI program. The research design of study subjects from the NHIRD is shown in Figure 1.

There are two types of studies (population based database analysis and clinical IgE and CAP detection) in the research. For data based study, the identification numbers of individuals in the NHRI databases (Taiwan) were encrypted to protect the privacy of the individuals, this part of study was approved to exempt from full review by the Institution Review Board of Kaohsiung Chang Gung Memorial Hospital; For clinical allergic factors detection, Institution Review Board of Kaohsiung Chang Gung Memorial Hospital approved this part of IgE and CAP studies. Design of work and final report conformed to the Declaration of Helsinki. Parents or guardians of these children sign written informed consent forms for this study.

### 2. Study population

We included patients aged 7∼15 years who had more than two ambulatory care visits or one hospital admission with any diagnosis of BA (ICD-9-CM codes 493.X), or AD (ICD-9-CM codes 691 and 691.8), or AR (ICD-9-CM codes 477.0, 477.1, 477.2, 477.8, and 477.9) from a sample of one million subjects because only one visit for outpatients might not have exactly confirmed these three diseases. In this study, we tested the correlation between these three allergic diseases with the month of birth of schoolchildren. We added the subject's gender, urbanization level, and residence region to our regression model to adjust our odds ratios (ORs).

Although 40% of children usually have asthma-like symptoms that commence at a preschool age, it is difficult to make a definite diagnosis of asthma in these children who may have transient wheezes or non-atopic wheezes [16], [17]. Therefore, we only enrolled schoolchildren who were aged 7∼15 years in 2010, then further classified schoolchildren into three age groups (7∼9, 10∼12, and 13∼15 years) to explore the associations between the season of birth and subsequent allergic diseases.

### 3. IgE level with asthma risk analysis

To identify associations of the month of birth with serum total IgE levels and sensitization (CAP), we collected blood samples from children in a cohort study as previously described [18]–[22]. In total, 987 children were recruited, and blood samples from the children were collected at 6 months old. Children's IgE data were collected during their visits to the Obstetric Clinic at Chang Gung Memorial Hospital, Kaohsiung, from September 1999 to December 2004. Concentrations of total IgE and CAP were measured using a Pharmacia CAP fluoroimmunoassay (Pharmacia and Upjohn Diagnostics AB, Uppsala, Sweden) following the manufacturer's instructions. Two common inhaled allergens (Dermatophagoides pteronyssinus (Derp) and cockroach), and four common food allergens (egg white, milk protein, shrimp, and peanut), which have prevalence of sensitization greater than 5% in the study country were included to assess allergen sensitization. CAP 1+ was defined as any specific IgE level of >0.35 kU/L, and CAP 2+ was defined as any specific IgE level of >0.7 kU/L.

### 4. Meteorological data

Meteorological data were obtained from the Taiwan Central Weather Bureau (CWB). According to the Taiwan CWB, the four seasons are defined by their characteristics of the average monthly ambient temperature, relative humidity, atmospheric pressure, rainfall, hours of sunshine, and maximum and minimum temperatures. Spring is February to April, summer is May to July, autumn is August to October, and winter is November to January.

### 5. Statistical analysis

Statistical analyses were conducted using SPSS software vers. 18.0 (SPSS, Chicago, IL, USA). All data were analyzed using a Chi-squared (Χ^2^) test for differences in the prevalence of related allergic diseases. ORs and 95% confidence intervals (CIs) were computed with the logistic regression analysis after taking the confounding variables (subjects' gender, urbanization level, and geographic region) into account. In this study, spring (February to April) was used as the base season to which the other seasons were compared for prevalence. Significance was set at *p*<0.05.

IgE of 6-month-old infants were analyzed in a logarithmic form. CAP+ and CAP2+ are expressed as percentages. An analysis of variance (ANOVA) test was used to compare allergic factors in different seasons, and *p* values of <0.05 were considered statistically significant. In addition, because IgE reflects allergic conditions and type I hypersensitivity, it was not normally distributed. We therefore classified IgE data into two groups: normal and high groups. Normal IgE was defined as a maternal IgE level of <100 kU/L, and a level in 6-month-old infants IgE of <20 kU/L, whereas high IgE was defined as a maternal IgE level of >100 kU/L, and a 6-month-old level of >20 kU/L. We used a logistic regression analysis to compute the ORs and 95% CIs after adjusting for maternal IgE, and *p* values of<0.05 were considered statistically significant.

## Results

### 1. Month of birth and prevalences of allergic diseases

**Figure 2:**
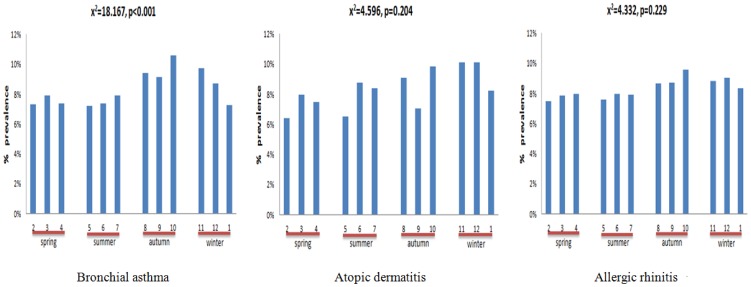
Month of Birth and the Prevalence of Allergic Diseases in schoolchildren.

We examined correlations between the month of birth and allergic diseases including BA, AD, and AR. As shown in Figure 2, there was a statistically significance correlation in the prevalence of BA with month of birth (Χ^2^ = 18.167, *p*<0.001). The fewest schoolchildren with BA were born in May (7.21%, OR: 0.91, CI: 0.80∼1.03), and the most were born in October (10.59%, OR: 1.16, CI: 1.05∼1.30). Moreover, neither the same tendency nor significant associations were found between the prevalence of AD (Χ^2^ = 4.6, *p* = 0.204) or AR (Χ^2^ = 4.3 *p* = 0.229) and the month of birth (Figure 2).

### 2. Birth season and the prevalence of BA

We further divided subjects into four groups based on the season of birth, and performed a logistic regression analysis to identify the association between birth season and BA (Table 1). The trend of sensitization across the four seasons showed that the rate in spring was lower than that in the other three seasons for the three allergic diseases (Figure 2). Therefore, we chose spring as the reference season to analyze moderate-to-severe BA in each season. The results showed that patients with BA who were born in autumn (August to October) had a 1.13-fold higher (95% CI: 1.05∼1.21) prevalence of BA than those born in spring after adjusting for subject's gender, urbanization level, and geographic region.

**Table 1:** Logistic regression analysis of season of birth and prevalence of bronchial asthma.

Season of birth		Odds ratio (95% confidence interval)	*p* value
Spring (Feb.∼Apr.)	(*n* = 24,928)	----	----
Summer (May∼July)	(*n* = 24,871)	1.00 (0.92∼1.08)	0.97
Autumn (Aug.∼Oct.)	(*n* = 27,526)	1.13 (1.05∼1.21)	**0.001** **
Winter (Nov.∼Jan.)	(*n* = 27,130)	1.04 (0.96∼1.12)	0.29

Adjustments were made for subject's gender, urbanization level, and geographic region.

** *p*<0.01.

### 3. Comparison of age and the prevalence of BA

To determine whether an effect of month of birth on the prevalence of BA could be observed from early or later childhood, we divided subjects into three age groups (7∼9, 10∼12, and 13∼15 years). Individuals aged 7∼9 and 13∼15 years showed significant correlation with the prevalence of BA (OR: 1.16, 95% CI: 1.04∼1.29 for 7∼9 years group; OR: 1.23, 95% CI: 1.02∼1.45 for 13∼15 years group). Although differences for those aged 10∼12 years did not achieve significance, the same trend was noted (OR: 1.08, 95% CI: 0.95∼1.23) (Table 2).

**Table 2:** Logistic regression analysis of season of birth and prevalence of bronchial asthma in separate age groups: comparing other seasons with spring.

	7∼9 years (*n* = 30,482)		10∼12 years (*n* = 35,422)		13∼15 years (*n* = 38,551)	
Season of birth	OR (95% CI)	*p* value	OR (95% CI)	*p* value	OR (95% CI)	*p* value
Spring (Feb.∼Apr.)	----	----	----	----	----	----
Summer (May∼July)	1.00 (0.90–1.12)	0.94	1.01 (0.88–1.14)	0.94	0.99 (0.83–1.20)	0.99
Autumn (Aug.∼Oct.)	1.16 (1.04–1.29)	**0.006** **	1.08 (0.95–1.23)	0.19	1.23 (1.02–1.45)	0.03*
Winter (Nov.∼Jan.)	1.09 (0.98–1.21)	0.10	0.97 (0.86–1.11)	0.72	1.06 (0.88–1.27)	0.54

Adjustments were made for subject's gender, urbanization level, and geographic region.

* *p*<0.05;

** *p*<0.01.

OR, odds ratio; CI, confidence interval.

### 4. Birth season and allergic markers

To determine whether the effects of month of birth were correlated with allergic markers, expression levels of allergic markers in mothers and their children were collected. As shown in Table 3, a significant association between IgE levels in children and birth season (autumn) was found. In addition, a more-significant association between children with CAP and birth season was detected. Children who were born in autumn had a higher level of CAP (Table 3). Maternal IgE is an important factor that might influence the correlation between IgE levels in children and the birth season. Thus we further adjusted for maternal IgE in this study. As shown in Table 4, children born in autumn had a significantly higher risk of asthma than that those born in spring (OR: 1.82, 95% CI: 1.27∼2.63) (Table 4).

**Table 3:** Association between birth season and children's allergic factors at 6 months old.

Season	Log IgE (*n* = 987)	CAP+ (*n* = 969)	CAP2+ (*n* = 969)
Spring (Feb.∼Apr.)	1.11±0.52	12.9%	8%
Summer (May∼July)	1.14±0.54	13.1%	8.3%
Autumn (Aug.∼Oct.)	1.24±0.54	24.5%	16.2%
Winter (Nov.∼Jan.)	1.14±0.49	11.5%	7.0%
*p* value	**0.043**	**<0.001**	**0.004**

IgE, immunoglobulin E (kU/L); CAP, ImmunoCAP allergen.

**Table 4:** Logistic regression analysis for birth seasons in the normal and high immunoglobulin E (IgE) groups.

Season of birth	Normal IgE	High IgE	OR (95% CI)	*p* value
Spring (Feb.∼Apr.)	167 (66.3%)	85 (33.7%)	----	----
Summer (May∼July)	164 (63.6%)	94 (36.4%)	1.11 (0.77–1.61)	0.571
Autumn (Aug.∼Oct.)	130 (52.6%)	117 (47.4%)	1.82 (1.27–2.63)	**0.001** **
Winter (Nov.∼Jan.)	152 (66.1%)	78 (33.9%)	1.02 (0.69–1.48)	0.936

Adjustments were made for maternal IgE. Normal IgE: IgE<100 kU/L for maternal IgE and IgE<20 kU/L for a 6-month-old child; High IgE: IgE>100 kU/L for maternal IgE and IgE>20 kU/L for a 6-month-old child.

** *p*<0.01.

OR, odds ratio; CI, confidence interval.

## Discussion

It has been reported that children born during autumn and winter have a higher risk of eczema compared to those born in spring and summer. Similar findings were observed in a large-scale population study in Japan, in which children born in autumn showed the highest prevalence of AD [9]. Importantly, the same tendency was observed for the prevalence of AD in this study, but with less significance. Takashi et al. suggested that children born in autumn are exposed to dry weather which may lead to dry skin. It is possible that dry skin is an important non-allergic etiologic factor for AD [9]. In Taiwan, the autumn and winter are more humid than in Japan, and this may explain why the association between season of birth and AD was not seen more clearly.

In this study, the key findings were that BA was more prevalent in children (especially those aged 7∼9 years) born in autumn, and less prevalent in those born in the other seasons. Cold temperatures, and especially very cold spells, were shown to increase the risk of pediatric asthma, as cold temperatures may induce hypersecretion from bronchial epithelial cells [23]. On the basis of this hypothesis, we speculated that children who are born in autumn may be exposed to cold weather (winter season) in their first months of life. This exposure may be an important environmental factor that triggers allergic reactions. In agreement with this hypothesis, children born in the winter season may be exposed to warmer temperatures in their first months of life (in spring) so that the prevalence of asthma and the level of allergic markers were lower.

Infection with multiple viruses in children is associated with a risk of asthma. Previous studies reported that respiratory syncytial virus (RSV) accounted for the most cases (43.4%) and followed by human bocavirus (19.5%), human metapneumovirus (16.8%), and human rhinovirus (HRV) (12.4%) [24]. In addition, a long-term follow-up study indicated that HRV bronchiolitis was linked to a higher risk of wheezing compared to RSV bronchiolitis [25], [26]. Results from a cohort study (COAST) also indicated that HRV-related wheezing was significantly associated with the risk for asthma at age 6 years compared to patients who wheezed due to RSV [26]. Thus, viral infection is an important risk factor for airway obstruction and asthma in children. Results from a Taiwanese study indicated that RSV circulates in the community throughout the year with a peak in July to October [27]. Consistent with results from Huang et al., we found that children who were born in autumn (August to October) were associated with a significantly higher risk of asthma.

Our findings highlight the significant correlation between season of birth and the risk of childhood BA. However, there are limitations to this association study. For example, the study population was extracted from the NHIRD based on administrative claims data reported by physicians. Although we established criteria for outpatients and inpatients to improve the diagnostic accuracy, our criteria are difficult to recruit subjects with mild bronchial asthma and are not able to define moderate-to-severe BA. In addition, the mode of delivery may influence the risk of asthma in children especially those delivered by an emergency caesarean section [28]. In this study, we did not collect enough data to analyze the correlation between the mode of delivery and allergic diseases. Thus, more studies are needed to better understand underlying biological mechanisms that may link environmental risk factors and allergic diseases.

Despite these limitations, our results from the longitudinal data and large study population should decrease the selection biases inherent in the database. In addition, significant correlations between season of birth and allergic markers (IgE and CAP) were observed. The relationship between season of birth and asthma might be one of the environmental factors in this complex disease, and a greater understanding of plausible allergens will help decrease the incidence of BA in children's early life in the future.

## References

[1] Eder W, Ege MJ, von Mutius E (2006) The asthma epidemic. N Engl J Med 355: 2226–2235. 17124020 10.1056/NEJMra054308

[2] Tamari M, Tomita K, Hirota T (2011) Genome-wide association studies of asthma. Allergol Int 60: 247–252. 21681015 10.2332/allergolint.11-RAI-0320

[3] Hirota T, Takahashi A, Kubo M, Tsunoda T, Tomita K, et al (2011) Genome-wide association study identifies three new susceptibility loci for adult asthma in the Japanese population. Nat Genet 43: 893–896. 21804548 10.1038/ng.887 PMC4310726

[4] Cookson W, Moffatt M, Strachan DP (2011) Genetic risks and childhood-onset asthma. J Allergy Clin Immunol 128: 266–270; quiz 271–262. 21807248 10.1016/j.jaci.2011.06.026

[5] Ege MJ, Mayer M, Normand AC, Genuneit J, Cookson WO, et al (2011) Exposure to environmental microorganisms and childhood asthma. N Engl J Med 364: 701–709. 21345099 10.1056/NEJMoa1007302

[6] Wan YI, Shrine NR, Soler Artigas M, Wain LV, Blakey JD, et al (2012) Genome-wide association study to identify genetic determinants of severe asthma. Thorax 67: 762–768. 22561531 10.1136/thoraxjnl-2011-201262

[7] Zhang Y, Moffatt MF, Cookson WO (2012) Genetic and genomic approaches to asthma: new insights for the origins. Curr Opin Pulm Med 18: 6–13. 22112999 10.1097/MCP.0b013e32834dc532

[8] Carlsten C, Melen E (2012) Air pollution, genetics, and allergy: an update. Curr Opin Allergy Clin Immunol 12: 455–460. 22885891 10.1097/ACI.0b013e328357cc55

[9] Kusunoki T, Asai K, Harazaki M, Korematsu S, Hosoi S (1999) Month of birth and prevalence of atopic dermatitis in schoolchildren: dry skin in early infancy as a possible etiologic factor. J Allergy Clin Immunol 103: 1148–1152. 10359898 10.1016/s0091-6749(99)70191-0

[10] Society. AT (2000) What constitutes an adverse health effect of air pollution? Official statement of the American Thoracic Society. Am J Respir Crit Care Med 161: 665–673. 10673213 10.1164/ajrccm.161.2.ats4-00

[11] Clark NM, Gong ZM, Wang SJ, Lin X, Bria WF, et al (2007) A randomized trial of a self-regulation intervention for women with asthma. CHEST Journal 132: 88–97. 10.1378/chest.06-2539 17505047

[12] Barnett AG, Williams GM, Schwartz J, Neller AH, Best TL, et al (2005) Air Pollution and Child Respiratory Health A Case-Crossover Study in Australia and New Zealand. American Journal of Respiratory and Critical Care Medicine 171: 1272–1278. 15764722 10.1164/rccm.200411-1586OC

[13] Sigurs N, Bjarnason R, Sigurbergsson F, Kjellman B (2000) Respiratory syncytial virus bronchiolitis in infancy is an important risk factor for asthma and allergy at age 7. American journal of respiratory and critical care medicine 161: 1501–1507. 10806145 10.1164/ajrccm.161.5.9906076

[14] Sly PD, Kusel M, Holt PG (2010) Do early-life viral infections cause asthma? Journal of Allergy and Clinical Immunology 125: 1202–1205. 20304476 10.1016/j.jaci.2010.01.024

[15] (2006) Department of Health, R.O.C. Taiwan: Public Health Report. Taiwan.

[16] Martinez FD, Wright AL, Taussig LM, Holberg CJ, Halonen M, et al (1995) Asthma and wheezing in the first six years of life. The Group Health Medical Associates. N Engl J Med 332: 133–138. 7800004 10.1056/NEJM199501193320301

[17] Taussig LM, Wright AL, Holberg CJ, Halonen M, Morgan WJ, et al (2003) Tucson Children's Respiratory Study: 1980 to present. J Allergy Clin Immunol 111: 661–675; quiz 676. 12704342 10.1067/mai.2003.162

[18] Yang KD, Ou CY, Chang JC, Chen RF, Liu CA, et al (2007) Infant frequent wheezing correlated to Clara cell protein 10 (CC10) polymorphism and concentration, but not allergy sensitization, in a perinatal cohort study. J Allergy Clin Immunol 120: 842–848. 17716718 10.1016/j.jaci.2007.07.009

[19] Liu CA, Wang CL, Chuang H, Ou CY, Hsu TY, et al (2003) Prenatal prediction of infant atopy by maternal but not paternal total IgE levels. J Allergy Clin Immunol 112: 899–904. 14610477 10.1016/j.jaci.2003.08.030

[20] Yang KD, Chang JC, Chuang H, Liang HM, Kuo HC, et al (2010) Gene-gene and gene-environment interactions on IgE production in prenatal stage. Allergy 65: 731–739. 19968631 10.1111/j.1398-9995.2009.02260.x

[21] Yang KD, Ou CY, Hsu TY, Chang JC, Chuang H, et al (2007) Interaction of maternal atopy, CTLA-4 gene polymorphism and gender on antenatal immunoglobulin E production. Clin Exp Allergy 37: 680–687. 17456215 10.1111/j.1365-2222.2007.02698.x

[22] Kuo HC, Liu CA, Ou CY, Hsu TY, Yang KD (2010) Correlation between atopy and tuberculin/Candida skin test reactivity in a bacillus Calmette- Gue'rin-vaccinated cohort. Allergy 65: 1625–1626. 20560903 10.1111/j.1398-9995.2010.02427.x

[23] Guo Y, Jiang F, Peng L, Zhang J, Geng F, et al (2012) The association between cold spells and pediatric outpatient visits for asthma in Shanghai, China. PLoS One 7: e42232. 22848748 10.1371/journal.pone.0042232 PMC3404967

[24] Chen Y-W, Huang Y-C, Ho T-H, Huang C-G, Tsao K-C, et al (2012) Viral etiology of bronchiolitis among pediatric inpatients in northern Taiwan with emphasis on newly identified respiratory viruses. Journal of Microbiology, Immunology and Infection 10.1016/j.jmii.2012.08.012 PMC7105232 23040235

[25] Kotaniemi-Syrjanen A, Vainionpaa R, Reijonen TM, Waris M, Korhonen K, et al (2003) Rhinovirus-induced wheezing in infancy–the first sign of childhood asthma? J Allergy Clin Immunol 111: 66–71. 12532098 10.1067/mai.2003.33 PMC7112360

[26] Jackson DJ, Gangnon RE, Evans MD, Roberg KA, Anderson EL, et al (2008) Wheezing rhinovirus illnesses in early life predict asthma development in high-risk children. Am J Respir Crit Care Med 178: 667–672. 18565953 10.1164/rccm.200802-309OC PMC2556448

[27] Huang YC, Lin TY, Chang LY, Wong KS, Ning SC (2001) Epidemiology of respiratory syncytial virus infection among paediatric inpatients in northern Taiwan. Eur J Pediatr 160: 581–582. 11585085 10.1007/s004310100803

[28] Almqvist C, Cnattingius S, Lichtenstein P, Lundholm C (2012) The impact of birth mode of delivery on childhood asthma and allergic diseases–a sibling study. Clin Exp Allergy 42: 1369–1376. 22925323 10.1111/j.1365-2222.2012.04021.x PMC3564396

